# Assessing the Performance of a Novel Stool-Based Microbiome Test That Predicts Response to First Line Immune Checkpoint Inhibitors in Multiple Cancer Types

**DOI:** 10.3390/cancers15133268

**Published:** 2023-06-21

**Authors:** Irina Robinson, Maximilian Johannes Hochmair, Manuela Schmidinger, Gudrun Absenger, Martin Pichler, Van Anh Nguyen, Erika Richtig, Barbara Margaretha Rainer, Leyla Ay, Christian Jansen, Cátia Pacífico, Alexander Knabl, Barbara Sladek, Nikolaus Gasche, Arschang Valipour

**Affiliations:** 1 Department of Respiratory and Critical Care Medicine, Karl Landsteiner Institute for Lung Research and Pulmonary Oncology, Klinik Floridsdorf, Vienna Healthcare Group, 1210 Vienna, Austria; maximilian.hochmair@gesundheitsverbund.at (M.J.H.); leyla.ay@gesundheitsverbund.at (L.A.); arschang.valipour@gesundheitsverbund.at (A.V.); 2 Department of Urology, Comprehensive Cancer Center, Medical University of Vienna, 1090 Vienna, Austria; manuela.schmidinger@meduniwien.ac.at; 3 Department of Internal Medicine, Division of Oncology, Medical University of Graz, 8036 Graz, Austria; gudrun.absenger@medunigraz.at (G.A.); martin.pichler@medunigraz.at (M.P.); 4 Department of Dermatology, Medical University Innsbruck, 6020 Innsbruck, Austria; v.nguyen@tirol-kliniken.at; 5 Department of Dermatology, Medical University of Graz, 8036 Graz, Austria; erika.richtig@medunigraz.at (E.R.); barbara.rainer@medunigraz.at (B.M.R.); 6 Biome Diagnostics GmbH, 1200 Vienna, Austria; christian.jansen@biome-dx.com (C.J.); catia.pacifico@biome-dx.com (C.P.); alexander.knabl@biome-dx.com (A.K.); barbara.sladek@biome-dx.com (B.S.); nikolaus.gasche@biome-dx.com (N.G.)

**Keywords:** immunotherapy, PD-L1, gut microbiome, microbiome profiling, next generation sequencing

## Abstract

**Simple Summary:**

Our study evaluated the use of the intestinal microbiome as a prognostic marker that seems to modulate response to immune checkpoint inhibitor (ICI) treatment in patients with late-stage non-small cell lung cancer (NSCLC), renal cell carcinoma (RCC), and metastatic melanoma. BiomeOne^®^, a stool microbiome-based algorithm, was able to classify patient samples according to the likeliness of achieving clinical benefit of ICI before treatment initiation by identifying an immunotherapy-favorable microbiome signature, outperforming the standard PD-L1 expression test. This study has additionally shed new light on the intestinal microbiome signature associated with the occurrence of irAEs, paving way to larger studies to validate and expand the current knowledge. Lastly, robust, easy-to-use, and non-invasive microbiome-based diagnostics hold promising potential for oncology, and further work should aim to expand these applications to other cancer types and microbiome-centered interventions, such as fecal microbiota transplantation (FMT), moving one step forward towards the era of personalized medicine.

**Abstract:**

The intestinal microbiome is by now an undebatable key player in the clinical outcome of ICI therapies. However, no microbiome profiling method to aid therapy decision is yet validated. We conducted a multi-centric study in patients with stage III/IV melanoma, NSCLC, or RCC receiving ICI treatment. The stool microbiome profile of 63 patients was analyzed with BiomeOne^®^, a microbiome-based algorithm that anticipates whether a patient will achieve clinical benefit with ICIs prior to therapy initiation. Classification of patient samples as Rs and NRs was achieved with a sensitivity of 81% and a specificity of 50% in this validation cohort. An ICI-favorable response was characterized by an intestinal microbiome rich in bacteria such as *Oscillospira* sp., Clostridia UCG-014, *Lachnospiraceae* UCG-010 sp., *Prevotella copri*, and a decrease in *Sutterella* sp., Lactobacillales, and *Streptococcus* sp. Patients who developed immune-related adverse events (irAEs) had an overall increased microbial diversity and richness, and a stool microbiome depleted in *Agathobacter*. When compared with the programmed death-ligand 1 (PD-L1) expression test in the subcohort of NSCLC patients (*n* = 38), BiomeOne^®^ exhibited a numerically higher sensitivity (78.6%) in identifying responders when compared with the PD-L1 test (67.9%). This study provides an evaluation of BiomeOne^®^, the first microbiome-based test for prediction of ICI response, to achieve market authorization. Validation with further indications and expansion to other microbiome-based interventions will be essential to bring microbiome-based diagnostics into standard clinical practice.

## 1. Introduction

Supported by cumulative evidence gathered by pre-clinical and clinical studies, the involvement of the intestinal microbiome in the efficacy of immune checkpoint inhibitors (ICIs) targeting the programmed cell death protein 1 (PD-1) and cytotoxic T-lymphocyte-associated antigen 4 (CTLA-4) inhibitory receptors and their PD-1 ligand (PD-L1) is now undisputed [1,2,3,4,5,6,7,8,9,10,11]. Mice that received stool transplants from responders (Rs) to anti-PD-1 therapy had an improved response to ICI therapy, increased up-regulation of PD-L1 in the tumor microenvironment, improved antigen presentation, and augmented effector T cell function in both the periphery and the tumor microenvironment, when compared with those transplanted with the stool from non-responders (NRs) [12]. Microbiomes from Rs were previously shown to be characterized by an increased alpha diversity and a higher abundance of *Ruminococcaceae* and *Faecalibacterium* [12], suggesting that response to ICIs could be converted by active modulation of the host’s intestinal milieu, either through stool transplantation or oral supplementation with ICI-favorable bacteria [5,8].

Hence, the intestinal microbiome emerged as a potential profiling source for ICI outcome prediction and as a therapeutic target to modulate and improve clinical response. The idea that an easily accessible, non-invasive stool sample collection can provide an accurate predictive factor for ICI response was quite a sought-after topic of clinical research, with several studies describing an immunotherapy-favorable microbial signature associated with response in melanoma [1,2,4,7,8,10,12,13,14], renal cell carcinoma [5], and non-small cell lung cancer [3,6,11]. Studies investigating longitudinal effects of ICI therapies did not find any significant intestinal microbiome modifications, highlighting the fact that stool samples can be used as a predictive tool over the course of treatment [11,13]. Currently used biomarkers based on PD-L1 expression and tumor mutational burden have limited sensitivity and specificity, and often do not account for tumor heterogeneity [15], warranting the need for better predictors of ICI response for an efficient patient selection.

As ICIs became more popular as strategies to induce antitumor immunity, it also became obvious that only unraveling the underlying microbiome signature associated with therapy response would not suffice. Even a patient with a high likelihood of responding to ICI therapy can experience serious irAEs, resulting in a decreased overall benefit from the treatment. IrAEs are known to lead to the discontinuation of ICI treatment in up to 30% of patients [16,17]. These side effects can be observed in all the organ systems and can occur even months or years after treatment discontinuation [18]. When combined, PD-L1 and CTLA-4 treatments are known to lead to a high level of toxicity, with around 30–50% of the patients experiencing severe side effects [19,20]. Differences at the level of the intestinal microbiome between patients experiencing low or high levels or irAEs were previously reported [6], and ICI-associated colitis was reversed by fecal microbiota transplantation, indicating that ICI toxicity is also associated with the profile of the intestinal microbiome [21].

In this study, we conducted a multi-centric evaluation of BiomeOne^®^, a microbiome-based test that identifies ICI-favorable patterns in the microbial composition of baseline stool samples of cancer patients with advanced stage NSCLC, RCC, and metastatic melanoma. Our data showed that the use of an easy and cost-effective stool collection method, combined with 16S rRNA gene profiling, is an efficient method to predict the outcome of ICI therapy and represents a first step to implement microbiome diagnostics in personalized medicine approaches.

## 2. Materials and Methods

### 2.1. Patients and Inclusion/Exclusion Criteria

Patients between 18 and 90 years of age with advanced (stage III/IV) NSCLC (without a druggable target), RCC, and melanoma, treated at the Klinik Floridsdorf in Vienna and Medical Universities of Graz, Vienna, and Innsbruck between January 2021 and February 2022, who received ICIs as standard-of-care first line, therapy were enrolled in this study. All relevant study data were recorded throughout this period. All patients were recruited according to good clinical practices and under Austrian law, with approved protocols by local ethics commissions (EK Nr 20-126-VK, EK Nr 32-621 ex 19/20, EK Nr 1287/2020), EK Nr 1990/2020) before starting the recruitment process. The study was conducted according to the Declaration of Helsinki and all participants provided written informed consent for participation in this research. Only treatment-naive first-line patients were recruited (except for local radiotherapy). Patients had to be eligible for cancer immunotherapy, physically and mentally able to understand instructions, and able to collect and send the stool samples. Excluded were patients who received systemic antibiotic treatment up to 30 days before treatment initiation. Brain metastasis was not an exclusion criterion.

### 2.2. Study Design and Treatments

All patients fulfilled the clinical requirements to initiate ICI therapy and received either anti-PD-1/PD-L1 treatment or anti-CTLA-4 in combination with anti-PD-1 treatment. Clinical information, including age, gender, cancer type and stage, treatment type, and occurrence and severity of irAEs were documented in the case report form (Clinspire^®^ Version 2019-12-10) and summarized in Table 1. The outcome of ICI therapy was recorded as complete response (CR), partial response (PR), stable disease (SD), and progressive disease (PD) based on clinical and radiological response evaluated by the treating physician. Patients were defined as responders if a partial or complete response was achieved (Rs). Patients with stable disease and progressive disease were classified as non-responders (NRs). Incidence and severity of irAEs were classified according to Common Terminology Criteria for Adverse Events (CTCAE, v4.03). CTCAE allows the classification of irAEs between grades 1 and 5, with grade 1 representing mild irAEs, grade 2 representing moderate irAEs, grade 3 representing severe irAEs, grade 4 representing life-threatening irAEs, and grade 5 representing death. Patients were further divided in two groups: those who did not experience irAEs and those who did (≥grade 1). When available, blood parameters and tumor specific markers, specifically PD-L1, were recorded.

### 2.3. Questionnaire Data

To investigate the role of potential covariates, further information regarding ethnicity, method of delivery (natural or C-section), diet, Bristol scale classification of stool, alcohol consumption, smoking habits, gastrointestinal perturbations, allergies, and rating of stress level, general health status, and psychological well-being of the patients was collected through a self-reported survey.

### 2.4. Sample Collection

Patient fecal samples were collected at baseline, prior to ICI treatment initiation, and at week 12 of treatment. Stool samples were collected by the patients themselves using an at-home Norgen stool collection kit (Norgen Biotek Corp., Thorold, ON, Canada). The sampling tubes included a preservation solution for stabilizing the microbial composition at room temperature during transport. Samples were returned to the laboratory for analysis via postal service and immediately stored at −20 °C upon arrival.

### 2.5. DNA Extraction and Sequencing

Microbial DNA was extracted on the KingFisher FLEX (Thermo Scientific, Waltham MA, USA) using the innuPREP AniPath DNA/RNA Kit 2.0-KFFLX (IST Innuscreen, Berlin, Germany) according to the manufacturer’s instructions. Quality and quantity of the DNA was assessed using a NanoDropTM 2000c spectrophotometer (Thermo Scientific) and fluorometrically with the Quant-it TM Pico Green TM dsDNA Assay Kit (Invitrogen by Thermo Scientific) on the SpectraMax M2 (Molecular Devices, San Jose, CA, USA). Amplicon sequencing with barcoded primers 341F (5′-CCTACGGGNGGCWGCAG-3′) and 806R (5′-GGACTACHVGGGTATCTAATCC-3′) flanking the V3–V4 hypervariable region of the eubacterial 16S rRNA gene was performed at the Medical University of Vienna. Briefly, approximately 50–1000 ng of genomic DNA per each sample was used for library construction. Sequencing was performed on an Illumina MiSeq platform using a 2 × 300 bp paired-end reads approach. Libraries were sequenced to a uniform high-depth targeting 50,000 paired-ends reads.

### 2.6. Bioinformatic Analysis

Raw reads were quality filtered and denoised with DADA2 v1.18.0, resulting in high-quality sequences. Taxonomy assignment of each unique amplicon sequence variants (ASV) was performed using the SILVA database v138. Samples were rarefied to 20,000 reads per sample. ASV table, taxonomy, and metadata were imported into a TreeSummarizedExperiment using the mia package v1.3.23. Microbiome diversity was assessed using the alpha diversity metrics number of observed ASVs, Shannon diversity, and Inverse Simpson. Distance between samples was measured based on Jaccard and Aitchison distances, using centered log-ratio (clr)-transformed values.

### 2.7. BiomeOne^®^ Response Prediction

To predict the probability of response to ICI therapy, the microbiome-based stool test BiomeOne^®^ (Biome Diagnostics, Vienna, Austria) was used. BiomeOne^®^ was developed by training several machine learning models on microbiome profiles from >10,000 of stool microbiome samples from both healthy individuals and cancer patients obtained from the Biome Diagnostics proprietary database and partner studies. When available, information such as sex, age, microbial richness, and diversity metrics was also provided as a feature. Several models (e.g., random forest, logistic regression, k-nearest neighbor, support vector machine) were used to classify Rs and NRs based on their microbiome profiles, both using clr-transformed and total sum normalized features at ASV, species and genus level using the python library scikit-learn v.0.24.2. Feature selection was performed using an autoencoder, SelectKBest, MaAsLin2, principal component analysis (PCA), and uniform manifold approximation and projection (UMAP). Model performance was assessed using stratified 5-fold cross-validation. Thus, 5 subsets were constructed by randomly dividing the overall dataset. Each of the 5 subsets was subsequently set as the testing set, with the remaining 4 subsets as training sets. The average of all 5 replicates was used to evaluate the performance of the models using the area under the precision-recall curve (AUPRC), F1, Matthews correlation coefficient (MCC), sensitivity, specificity, positive predictive values (PPV), and negative predictive values (NPV). The best model identified was then selected and used to create the medical product BiomeOne^®^, which yielded a prediction sensitivity of 87% on a trained dataset of the classifier. The aim of this study was the assessment of the performance of the algorithm, using this multi-centric cohort composed by NSCLC, RCC, and melanoma patients as a validation dataset. BiomeOne^®^ outputs a probability of response after recognizing specific patterns in the microbiome composition. If a given sample had a probability of response >50%, the patient was highly likely to benefit from an ICI therapy and was classified as a responder [22].

### 2.8. Statistical Analyses

All statistical analyses were performed in R v4.1.3. From an initial estimation of 90 patients, 80 were recruited and successfully enrolled in this study and the cohort size was consistent with previous publications found in the literature [7,8]. Fisher’s exact test was used to inspect differences between R and NR at the level of categorical patient characteristics and questionnaire data. Tetrachoric correlations between binary variables were conducted using the package psych v2.2.5. To test for differences between two groups, the Mann–Whitney U test was used, while multiple group comparisons were carried out via the Kruskal–Wallis test. Permutational multivariate analysis of variance (PERMANOVA) was calculated with the adonis2 function and used to assess significant beta-diversity differences between groups. The results were visualized using a principal coordinates analysis (PcoA) plot. Confounding factor analysis was performed using metadeconfoundR v0.2.8. Covariates such as patient ID, age, sex, type of therapy, form of cancer, cancer stage, and presence/absence of irAEs were included in the analysis to obtain a microbial signature exclusively associated with response to ICIs. Differential abundance (DA) analysis was performed at the species level, combining the output of the R packages ALDEx2 v1.26.0 (Welch’s *t* and Wilcoxon rank test) and MaAsLin2 v1.8.0. A *p*-value < 0.05 was considered statistically significant. For multiple pairwise comparisons, the Benjamini–Hochberg correction was used to adjust the *p*-value.

## 3. Results

### 3.1. Patients’ Demographics

From the 80 recruited patients, only 65 provided at least a baseline stool sample (Supplementary Figure S1). Thus, our cohort included 42 patients with NSCLC, 16 patients with RCC and 7 patients with metastatic melanoma (Table 1). Recruitment was balanced with slightly more males enrolled than females (*n* = 38, 58.46%). The mean age was 66.57 ± 8.78 years. The mean body mass index (BMI) of the 61 patients (Supplementary Table S1) at baseline was 25.87 ± 4.66 kg/m^2^. Our cohort included patients with mainly stage IV cancer (76.92%). Throughout the entire duration of the trial, 11 patients were treated with ICI monotherapy, 42 with combination therapy, 6 with mono- and combination therapy, 4 with combination therapy and chemotherapy, and 2 with mono-, combination and chemotherapy. Only three patients (4.62%) received anti-PD-1/CTLA-4 ICI combination therapy. A total of 112 stool samples were collected before (*n* = 65) or after ICI treatment, at week 12 (*n* = 47). Forty-three patients were classified as Rs and twenty-two patients were NRs. No significant differences regarding sex, age, cancer type, and stage were found between Rs and NRs at baseline. There were no significant differences between stool frequency (*p* = 0.30) or consistency (*p* = 0.75) or any of the other self-reported patients’ characteristics and lifestyle factors between R and NR (Supplementary Table S2). Approximately 67.69% of the patients enrolled in this study experienced irAEs (≥grade 1), which seemed to impact the responsiveness to ICI therapy, according to Fisher’s exact test (*p* < 0.01). Interestingly, tetrachoric correlation analysis further confirmed an association between irAEs and the outcome of ICI therapy (r = 0.75).

### 3.2. Longitudinal Influence of ICI Treatment on the Intestinal Microbiome

**Figure 1:**
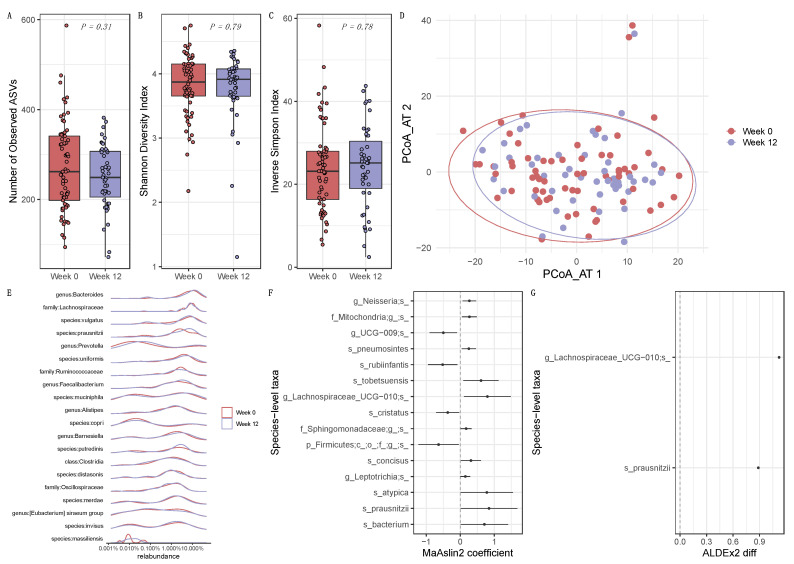
Comparison between baseline and therapy. (**A**–**C**) Number of observed ASVs, Shannon diversity index, and the Inverse Simpson index, respectively, showing no difference in alpha diversity between baseline (week 0) and week 12 (*p* > 0.05). (**D**) Principal coordinate analysis (PCoA) using the Aitchison distance matrix of all stool samples included in this study, showing no clear clustering regarding the time point of analysis. (**E**) Relative abundance plot of the top 20 species across all 112 sequenced samples. (**F**,**G**) Differential abundance analysis between week 0 and week 12 using MaAsLin2 and ALDEx2, respectively. Only results with a *p* < 0.05 are considered statistically significant. The reference level used was week 12. Negative coefficients indicate the microbial species that decreased in week 12, while positive coefficients indicate microbial species that increased.

To estimate the impact of ICI therapy on the intestinal microbiome, differences at the level of alpha diversity, species beta diversity, and composition were evaluated. No significant differences between baseline and week 12 samples were found for the number of observed ASVs (*p* = 0.31, Figure 1A), Shannon index (*p* = 0.79, Figure 1B), and the Inverse Simpson (*p* = 0.78, Figure 1C). No evident effect of ICI treatment on the microbial community structure was detected using PERMANOVA on both Jaccard (*p* = 0.95) and Aitchison (*p* = 1.0, Figure 1D) distance matrices. The top 20 most abundant microbial species are given in Figure 1E. However, compositional changes between baseline and week 12 were found. Faecalibacterium prausnitzii and a species belonging to the Lachnospiraceae UCG-010 genus were found to increase after therapy (*p* < 0.05), according to MaAsLin2 (Figure 1F) and ALDEx2 (Figure 1G).

### 3.3. Prediction of Clinical Outcome of ICI Therapy Using the Stool-Based Microbiome Test BiomeOne^®^ at Baseline

Out of the 65 baseline stool samples available, a total of 63 were screened with the microbiome-based ICI response prediction test BiomeOne^®^ and compared with clinical response. Of these, 42 patients were classified as Rs, and 21 as NRs, based on the clinical outcome (Table 2). From the 42 patients classified as Rs, BiomeOne^®^ successfully identified 34 as very likely to benefit from ICI therapy (overall sensitivity of 81% and specificity of 52%). In the NR group, 11 out 21 patients that were unlikely to benefit from the ICI therapy were successfully detected. This represents a positive predictive value (PPV) of 77%, and a negative predictive value (NPV) of 58%.

### 3.4. Baseline Compositional Differences May Drive Clinical Response

**Figure 2:**
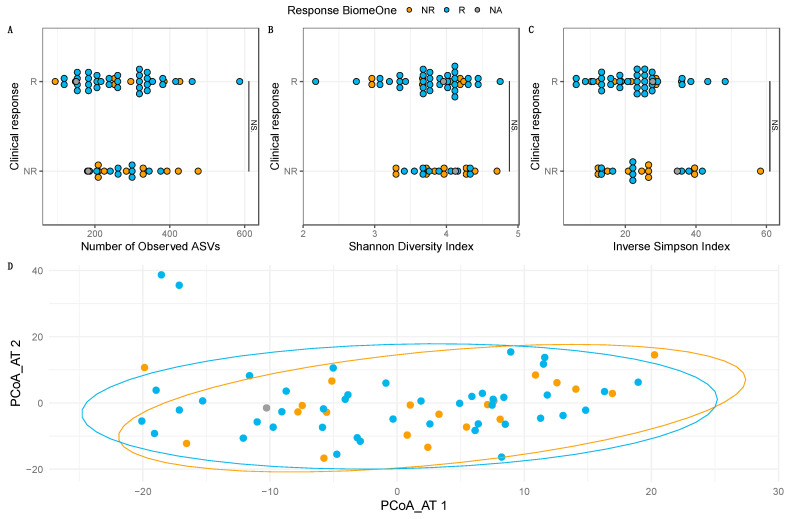
Comparison between Rs and NRs, at the level of alpha diversity (**A**–**C**) and beta diversity (**D**). Rs and NRs are grouped according to the clinical response and colored based on the BiomeOne^®^ model prediction. No significant differences were found between Rs and NRs regarding the number of observed ASVs, Shannon diversity index, and the Inverse Simpson index (*p* < 0.05, NS). PERMANOVA did not detect any significant differences regarding the Aitchison distances (*p* < 0.05) between stool samples of Rs and NRs.

Differences between Rs and NRs classified according to BiomeOne^®^ and clinical response were investigated. No detectable changes between groups were found for the number of observed ASVs (*p* = 0.51, Figure 2A), Shannon diversity index (*p* = 0.64, Figure 2B), and the Inverse Simpson (*p* = 0.75, Figure 2C), according to the model classification. No significant differences were found regarding beta diversity for both Jaccard (*p* = 0.09) and Aitchison (*p* = 0.10, Figure 2D) distance matrices, according to PERMANOVA. These results were consistent when stratifying the samples according to clinical response, indicating that the overall microbiome richness and diversity did not differ between Rs and NRs (*p* > 0.05). Additionally, no changes in alpha and beta diversity could be found between cancer types (*p* < 0.05).

Potential sources of variation that could obscure the microbial signature associated with ICIs were selected as covariates of interest for deconfounding analysis. Patient ID, age, sex, type of therapy, form of cancer, cancer stage, and presence/absence of irAEs were given as an input. From the 469 microbial species investigated, no significant associations were found between clinical response and any other covariate. Rs identified by BiomeOne^®^ had a stool microbiome enriched with *Oscillospira* sp., (order) Clostridia UCG-014, Prevotella copri, and *Lachnospiraceae* UCG-010 sp. According to ALDEx2 (Supplementary Table S3). *Sutterella* sp., *Streptococcus* sp., and (order) Lactobacillales were decreased in Rs. MaAsLin2 (Supplementary Table S4) identified an additional 14 other bacteria associated with Rs/NRs, as predicted by the model. These included *Paludicola* sp., *Shuttleworthia* sp., (family) Clostridiaceae, Parasutterella secunda, Bacteroides finegoldii, Romboutsia sedimentorum, *Gemella sanguinis*, CHKCI002 sp., Bacteroides coprocola, *Methanobrevibacter* sp., and Bifidobacterium bifidum, all associated with NRs. Lachnospiraceae NC2004 group sp., Coprobacillus cateniformis, and (family) UCG-010 increased in Rs. When analyzing the differential abundance of all microbial species classified according to the clinical outcome, *Sutterella* sp., *Eubacterium halii* group sp., and *Haemophilus* sp. were all significantly decreased in Rs, according to both ALDEx2 and MaAsLin2 (Supplementary Tables S5 and S6).

### 3.5. BiomeOne^®^ Exhibited Higher Sensitivity in Predicting Response Than the PD-L1 Expression Test

**Figure 3:**
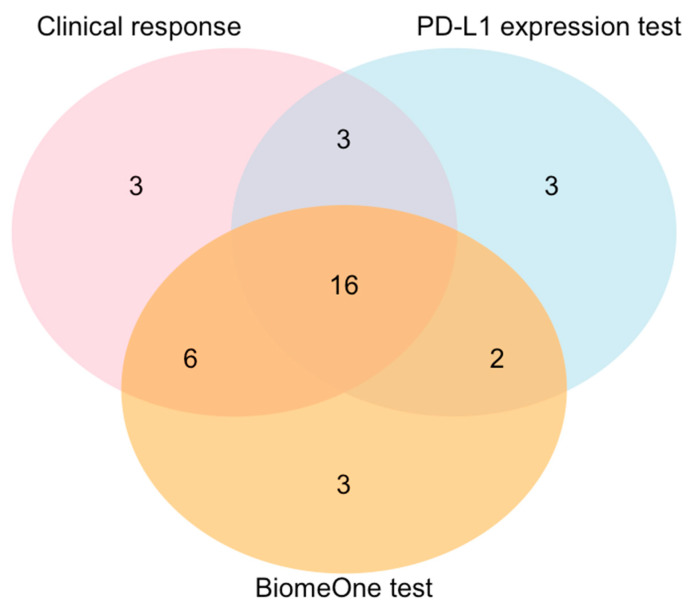
Venn diagram with the population of Rs identified in the NSCLC subcohort (*n* = 38), according to clinical outcome, PD-L1 expression test (>1%), and BiomeOne^®^. All classification methods concordantly identify 16 out of 28 Rs.

A subset of 38 NSCLC patients (Rs *n* = 28, NRs *n* = 10) for which both BiomeOne^®^ predictions and PD-L1 expression data were available were compared regarding specificity and sensitivity [23]. BiomeOne^®^ identified a total of 27 Rs, while the PD-L1 assay identified a total of 24 (with PD-L1 positivity threshold >1%). When combined, both methods concordantly identified 16 out of the 28 responders classified according to their clinical outcome (Figure 3). BiomeOne^®^ exhibited an overall sensitivity of 78.6% (95% CI 63.3, 93.8) and a specificity of 50.0% (95% CI 19.0, 80.1), while the PD-L1 assay achieved a lower sensitivity, of 67.9% (95% CI 50.6, 85.1), despite achieving a comparable specificity (50.0%, 95% CI 19.0, 80.1).

### 3.6. Occurrence/Severity of irAEs Depends Heavily on the Baseline Intestinal Microbiome Composition

**Figure 4:**
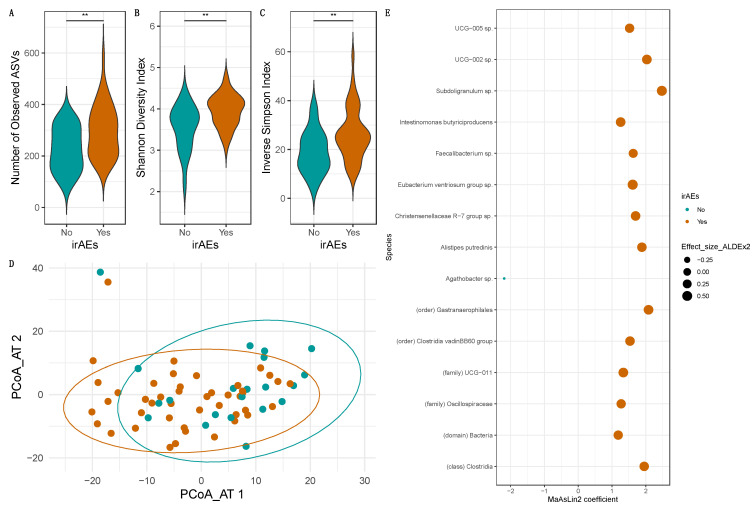
Comparison between patients experiencing irAEs or no irAEs, at the level of alpha diversity (**A**–**C**), beta diversity (**D**) and differential abundance (**E**). Significant differences between patients developing irAEs and patients without occurrence of irAEs were found for the number of observed ASVs, Shannon diversity index, and the Inverse Simpson index (*p* < 0.01, **). Development of irAEs was overall associated with a higher richness and diversity of the stool microbiome. This is consistent with the presence of two clusters separating both groups in the Aitchison distance PCoA (*p* = 0.05). DA microbial species found by both MaAsLin2 and ALDEx2 are summarized in E.4. Discussion.

In our cohort, patients that experienced irAEs had an increased microbial diversity and richness at baseline than those that reported no irAEs, as evidenced by an increased number of observed ASVs (*p* < 0.01, Figure 4A), and a higher Shannon (*p* < 0.01, Figure 4B) and the Inverse Simpson (*p* < 0.01, Figure 4C) indices. Significant changes in beta diversity were observed on both the Aitchison (*p* = 0.05, Figure 4D) and Jaccard (*p* = 0.04) distance matrices.

ALDEx2 identified a total of 17 DA species, while MaAsLin2 detected a total of 97 (Supplementary Table S3). The number of overlapping DA species between both methods was 15 (Figure 4E). Patients who experienced irAEs had a higher abundance of Intestinimonas butyriciproducens, Alistipes putredinis, and unclassified species belonging to the genera Eubacterium ventriosum group, Christensenellaceae R-7 group, Faecalibacterium, Subdoligranulum, [Oscillospiracea] UCG-002, and [Oscillospiracea] UCG-005. Additionally, six other unclassified bacterial species at higher taxonomic levels were also increased in these patients (*p* < 0.05). *Agathobacter* sp. was found to be positively associated with the absence of irAEs (*p* < 0.02).

In this study, we were able to confirm the association between the intestinal microbiome composition and ICI therapy outcome in patients with advanced stage NSCLC, RCC and melanoma using BiomeOne^®^, based on the patient’s stool microbiome at baseline. Additionally, we conducted a thorough assessment of the intestinal microbiome changes induced by ICI treatment and explored a possible link between baseline microbiome and the development of irAEs.

Anti-PD-1/PD-L1 and anti-PD-1/CTLA-4 therapy had minimal impact on the richness, diversity, and species abundance of the intestinal microbiome of patients with advanced NSCLC, RCC, and melanoma, when comparing baseline and week 12 of treatment. Decreased microbiome diversity (dysbiosis) was reported in association with human diseases and inflammation. In the context of ICIs, the intestinal microbiome was previously reported to remain stable over the course of ipilimumab treatment, with no significant changes at the level of Shannon and Simpson diversity indices throughout the course of treatment [13]. Our data were consistent with these findings, as no indications of ICI-induced dysbiosis were detected after 12 weeks of treatment. However, at the species level, *F. prausnitzii* and *Lachnospiraceae* UCG-010 sp. were found to increase at week 12 when compared with baseline by both differential analysis methods. *F. prausnitzii*, a major butyrate producer contributing to the anti-inflammatory balance of the intestines [24,25], was found across several studies involving patients with metastatic melanoma [1,7,12,13]. This commensal bacterium was enriched in the microbiome of Rs to ipilimumab plus nivolumab treatment [1] and, in another study, was associated with overall PFS [7]. Gopalakrishnan et al. [12] observed a significant positive correlation between the relative abundance of Faecalibacterium and the frequencies of tumor CD8+ T cell infiltrate and peripheral CD8+ T cell and effector CD4+ T cell, as well as a positive association with peripheral cytokine profile for response to ICI treatment, highlighting a positive immunomodulatory effect of this bacterial genus. Thus, the increase in *F. prausnitzii* observed in our study between baseline and after 12 weeks of ICI therapy may be associated with an improvement of the overall immune cell landscape of the patients after treatment initiation.

Even though several studies described that changes in the microbial composition at baseline could determine the success of ICI treatment, none of these observations were translated into a clinical application. To the best of our knowledge, BiomeOne^®^ is the first stool test that analyzes the microbiome composition of the individual patient before therapy initiation that entered the market and achieved market authorization in Europe. This test allows an easy and discreet stool collection at home and can be ordered by oncologists and physicians to support therapy decision. Despite the differences in geographic location, genetic background, study characteristics, laboratory methodologies, and sequencing approaches, a model classifying Rs and NRs in a training dataset with >10,000 samples was developed with a sensitivity of 87%. When using this multi-centric study as a validation cohort, we were able to identify Rs and NRs with a sensitivity, specificity, PPV, and NPV of 81%, 52%, 77%, and 58%, respectively.

Differences between Rs and NRs classified according to the BiomeOne^®^ model were further investigated to obtain a deeper insight at the microbial compositional patterns identified by the test in our study cohort. Previous observations reported no changes in alpha and beta diversity between Rs and NRs at baseline, before treatment initiation [2,14]. Our results confirmed these data, as the response measured by either clinical outcome or based on BiomeOne^®^ prediction did not seem to be associated with the number of observed ASVs, nor Shannon and Inverse Simpson diversity indices. Additionally, when visualizing the beta diversity through PCoA analysis of each sample collapsed at the species level, no evident clustering was observed separating Rs from NRs. However, not all reports align with these observations.

A link between microbial richness and PFS was previously found in melanoma patients [7], and a lower alpha diversity was consistent with a shorter OS [6]. Inconsistent results between studies could be due to intrinsic variability associated with the studies’ populations, sample size, or technical bias introduced by laboratory and bioinformatic methodologies. The same arguments are valid for the lack of coherent microbial signatures found across studies.

Changes on species-level composition between Rs and NRs were detected at baseline, with *Sutterella* sp. being enriched in NRs, independently of how they were classified. A previous study on melanoma revealed that progressive disease was associated with an increased abundance in *Sutterellaceae* spp. in the stool microbiome of these patients, together with an increased abundance of *Prevotella* spp., *Oscillibacter* spp., and *Alistipes* spp. [14]. In another cohort consisting of NSCLC patients responding to nivolumab treatment, *Sutterella* sp. was more abundant in patients with little clinical benefit [26]. In total, seven species were identified by ALDEx2 and fourteen others were identified by MaAsLin2 in our study, as being differentially abundant between Rs and NRs. Some bacteria increasing in Rs may trigger pro-inflammatory pathways, which, in the context of immunotherapy, would be an important asset in immune surveillance, by exacerbating the immune responses. Prevotella copri, found by both ALDEx2 and MaAsLin2 to increase in Rs in our study, was previously shown to increase in NSCLC patients responding to PD-1 blockade, and to possibly trigger proliferation of pro-inflammatory T cells [27].

In our study, confounding factors analysis did not detect any study-specific characteristics that could have obscured the microbial signature associated with ICI response. This is particularly relevant, as tumor type seems to have not impacted these results nor was significant when investigating differences at the level of alpha or beta diversity. Additionally, none of the self-reported characteristics of the patients or the sample (consistency and frequency) seemed to blur the microbial signature associated with ICI response. The intestinal microbiome seemed to impact the effectiveness of immunotherapy by modulating effector and suppressor immune cell populations through the production of microbe-derived metabolites, antigens, and pathogen-associated molecular patterns [28]. Therefore, to understand why the enrichment of specific microbes in the intestine prior beginning ICI therapy dictates treatment success, a deeper analysis of the crosstalk between intestinal microbiome and immune system must be undertaken.

Deciding whether to treat a NSCLC patient with either ICIs or combination therapies mainly relied on the measurement of the expression of the PD-L1 ligand in the tumor cells. However, accumulating evidence showed that PD-L1 alone does not predict response to ICIs accurately enough. Biomarkers or company diagnostics based on the intestinal microbiome signature of the patient stool led the research focus in recent years but only recently resulted in an approved product. Our study compared both PD-L1 and BiomeOne^®^ test performance in a subcohort of NSCLC patients, showing that BiomeOne^®^ can potentially outperform the PD-L1 expression test when identifying Rs. When used alone, PD-L1 is a rather weak biomarker [29], but a combination of biomarkers could potentially improve the detection of Rs and NRs. Consequently, BiomeOne^®^ represents an interesting alternative or addition to PD-L1 and may, thus, be a promising diagnostic tool to be used in combination with the PD-L1 expression test.

Previous research investigating the impact of the intestinal microbiome on irAEs did not find a difference in the bacterial diversity of patients who experienced irAE ≥ grade 2 [6] or ≥grade 3 [2] and those with non-severe irAE. However, specific irAEs, such as immune-related colitis, were shown to be associated with a decreased Shannon diversity index [13]. Our data showed that patients experiencing irAEs have a higher microbial diversity and richness of their stool microbiome, than those not developing irAEs. Interestingly, higher diversity and richness tended to be associated with health. In our study, out of the 44 patients that experienced irAEs, 23 were Rs while 21 were NRs. In contrast to previous reports, patients with irAEs were more predominant amongst NRs, with only one NR out of twenty-two not experiencing irAEs. Bacteria such as *Intestinimonas butyriciproducens*, *Alistipes putredinis*, unclassified species belonging to the genera *Eubacterium ventriosum* group, Christensenellaceae R-7 group, *Faecalibacterium*, *Subdoligranulum*, [Oscillospiracea] UCG-002, and [Oscillospiracea] UCG-005 were found to be associated with the development of irAEs in our study. *Agathobacter* sp. was previously reported to be associated with favorable objective response rate (ORR) and progression-free survival (PFS) > 6 months in a NSCLC cohort, while being overrepresented in patients with a more severe irAE profile [6]. In our cohort, a higher abundance of *Agathobacter* sp. was rather associated with the absence of irAEs, which was inconsistent with the previous findings. The lack of concordance between reports may, however, be cohort-specific [30,31]. Hence, larger, more diverse cohorts are needed to increase robustness of detection of such microbial signatures. Moreover, future studies should include functional profiling to further understand how microbial metabolism can impact the effectiveness of ICIs and development of irAEs.

Targeted 16S rRNA sequencing and metagenomic shotgun sequencing can provide robust results and accurate depictions of the profile of the intestinal microbiome [7]. The combination of these approaches with machine learning techniques are fundamental to handle large amounts of sequencing data and can create interpretable models for several medical applications. Based on this methodology, BiomeOne^®^ was able to identify a considerable proportion of Rs and NRs.

## 4. Conclusions

Even though this study brought added value to the pursuit of microbiome-based biomarkers, it was not without some limitations. First, we must acknowledge that a considerable proportion of patients did not comply with the study protocol, which underpowered the study. Second, the patient sample in the current report was enriched with NSCLC patients, which were the most recruited patient group among the three indications that we aimed to cover. Thus, generalizability and predictive capacity may not be equally distributed to the other cancer entities tested. Finally, we must admit that the relatively small patient sample may prevent robust conclusions, particularly in subsets and subcohorts. Nevertheless, we believe that our study highlighted the potential use of BiomeOne^®^ as a diagnostic tool in the scope of other therapeutic interventions, and its potential use as a tool to monitor the outcome of strategies that aim to modulate the intestinal microbiome, such as fecal microbiota transplantation, diet, or probiotic supplementation.

## Figures and Tables

**Table 1:** Baseline patient characteristics between Rs and NRs included in our study. Results are given in numbers (*n*) and respective %, except when stated otherwise. Fisher’s exact test was used to inspect differences between Rs and NRs at a significance level of 0.05.

		Total (*n* = 65)	R (*n* = 43)	NR (*n* = 22)	*p*-Value
**Sex**					
Male		38 (58.46)	24 (55.81)	14 (63.64)	0.60
Female		27 (41.54)	19 (44.19)	8 (36.36)	
**Age (mean ± SD, years)**		66.57 ± 8.78	65.38 ± 8.69	68.91 ± 8.67	0.12
**Cancer type**					
NSCLC	Stage III	11 (16.92)	10 (23.26)	1 (4.54)	0.07
	Stage IV	31 (47.69)	21 (48.84)	10 (45.45)	
RCC	Stage III	4 (6.15)	3 (6.98)	1 (4.54)	
	Stage IV	12 (18.46)	7 (16.28)	5 (22.73)	
Melanoma	Stage III	0 (0.00)	0 (0.00)	0 (0.00)	
	Stage IV	7 (10.77)	2 (4.65)	5 (22.73)	
**Treatment**					
Anti-PD-1/PD-L1		62 (95.38)	43 (100.00)	19 (86.36)	0.04
Anti-PD-1/CTLA-4		3 (4.62)	0 (0.00)	3 (13.64)	
**irAEs**					
No		21 (32.31)	20 (46.51)	1 (4.55)	<0.01
Yes (≥grade 1)		44 (67.69)	23 (53.49)	21 (95.45)	

**Table 2:** BiomeOne^®^ model predictions in 63 baseline stool samples, where 42 patients were classified as Rs, and 21 as NRs, based on the clinical outcome. A total of 34 out of 42 Rs were identified by the BiomeOne^®^ model, with a sensitivity, specificity, PPV, and NPV of 81%, 52%, 77%, and 58%, respectively.

	Rs (CR + PR)	NRs (SD + PD)	Total
BiomeOne result > 50%	34	10	**44**
BiomeOne result ≤ 50%	8	11	**19**
**Total**	**42**	**21**	**63**

## Data Availability

The 16S rRNA sequencing data were part of the proprietary biomarker development pipeline from Biome Diagnostics GmbH and can be made available upon reasonable request.

## Supplementary Materials

The following supporting information can be downloaded at: https://www.mdpi.com/article/10.3390/cancers15133268/s1, Figure S1: Flowchart of patients enrolled since study entry and details about inclusion and exclusion criteria. All analysis performed with this dataset are highlighted in orange and are accompanied by the respective number (n), which represents the total amount of patients included for each analysis step; Table S1: Questionnaire information obtained during baseline sample collection. Results are given in numbers (n) and respective %, except when stated otherwise. Fisher’s exact test was used to detect differences between Rs and NRs at a significance level of 0.05; Table S2: Questionnaire results regarding baseline sample information. Fisher’s exact test was used to detect differences between Rs and NRs at a significance level of 0.05; Table S3: Differentially abundant (DA) microbial species identified by ALDEx2 between Rs and NRs classified by the BiomeOne® algorithm. ALDEx2 uses the Wilcoxon Rank Sum test and Welch’s *t*-test to analyze clr-transformed variation in taxa between samples. A total of 7 DA species could be identified using this method; Table S4: Differentially abundant (DA) microbial species identified by MaAsLin2 between Rs and NRs classified by the BiomeOne® algorithm. MaAsLin2 performs general linear models on clr-transformed species abundance data, identifying a total of 21 DA taxa in our dataset; Table S5: Differentially abundant (DA) microbial species identified by ALDEx2 and MaAsLin2 between Rs and NRs classified according to clinical and radiological response. Reference level used for MaAsLin2 was the Rs group. Negative coefficients indicate the microbial species that decreased in Rs, while positive coefficients indicate microbial species enriched in Rs; Table S6: Differentially abundant (DA) microbial species identified by ALDEx2 and MaAsLin2 between patients experiencing (Yes) or not (No) immune-related adverse events (irAEs). Reference level used for MaAsLin2 was the Yes group. Negative coefficients indicate the microbial species that decreased in patients that experienced irAEs, while positive coefficients indicate microbial species that increased in this group.

## References

[1] Frankel A.E., Coughlin L.A., Kim J., Froehlich T.W., Xie Y., Frenkel E.P., Koh A.Y. (2017). Metagenomic Shotgun Sequencing and Unbiased Metabolomic Profiling Identify Specific Human Gut Microbiota and Metabolites Associated with Immune Checkpoint Therapy Efficacy in Melanoma Patients. Neoplasia.

[2] Andrews M.C., Duong C.P.M., Gopalakrishnan V., Iebba V., Chen W.-S., Derosa L., Khan A.W., Cogdill A.P., White M.G., Wong M.C. (2021). Gut microbiota signatures are associated with toxicity to combined CTLA-4 and PD-1 blockade. Nat. Med..

[3] Chau J., Yadav M., Ben Liu B., Furqan M., Dai Q., Shahi S., Gupta A., Mercer K.N., Eastman E., Abu Hejleh T. (2021). Prospective correlation between the patient microbiome with response to and development of immune-mediated adverse effects to immunotherapy in lung cancer. BMC Cancer.

[4] Spencer C.N., McQuade J.L., Gopalakrishnan V., McCulloch J.A., Vetizou M., Cogdill A.P., Khan A.W., Zhang X., White M.G., Peterson C.B. (2021). Dietary fiber and probiotics influence the gut microbiome and melanoma immunotherapy response. Science.

[5] Routy B., le Chatelier E., DeRosa L., Duong C.P.M., Alou M.T., Daillère R., Fluckiger A., Messaoudene M., Rauber C., Roberti M.P. (2018). Gut microbiome influences efficacy of PD-1–based immunotherapy against epithelial tumors. Science.

[6] Hakozaki T., Richard C., Elkrief A., Hosomi Y., Benlaïfaoui M., Mimpen I., Terrisse S., Derosa L., Zitvogel L., Routy B. (2020). The Gut Microbiome Associates with Immune Checkpoint Inhibition Outcomes in Patients with Advanced Non–Small Cell Lung Cancer. Cancer Immunol. Res..

[7] Peters B.A., Wilson M., Moran U., Pavlick A., Izsak A., Wechter T., Weber J.S., Osman I., Ahn J. (2019). Relating the gut metagenome and metatranscriptome to immunotherapy responses in melanoma patients. Genome Med..

[8] Matson V., Fessler J., Bao R., Chongsuwat T., Zha Y., Alegre M.-L., Luke J.J., Gajewski T.F. (2018). The commensal microbiome is associated with anti–PD-1 efficacy in metastatic melanoma patients. Science.

[9] Zheng Y., Fang Z., Xue Y., Zhang J., Zhu J., Gao R., Yao S., Ye Y., Wang S., Lin C. (2020). Specific gut microbiome signature predicts the early-stage lung cancer. Gut Microbes.

[10] Baruch E.N., Youngster I., Ben-Betzalel G., Ortenberg R., Lahat A., Katz L., Adler K., Dick-Necula D., Raskin S., Bloch N. (2020). Fecal microbiota transplant promotes response in immunotherapy-refractory melanoma patients. Science.

[11] Cascone T., William W.N., Weissferdt A., Leung C.H., Lin H.Y., Pataer A., Godoy M.C.B., Carter B.W., Federico L., Reuben A. (2021). Neoadjuvant nivolumab or nivolumab plus ipilimumab in operable non-small cell lung cancer: The phase 2 randomized NEOSTAR trial. Nat. Med..

[12] Gopalakrishnan V. (2018). Gut microbiome modulates response to anti–PD-1 immunotherapy in melanoma patients. Science.

[13] Chaput N., Lepage P., Coutzac C., Soularue E., Le Roux K., Monot C., Boselli L., Routier E., Cassard L., Collins M. (2017). Baseline gut microbiota predicts clinical response and colitis in metastatic melanoma patients treated with ipilimumab. Ann. Oncol..

[14] McCulloch J.A., Davar D., Rodrigues R.R., Badger J.H., Fang J.R., Cole A.M., Balaji A.K., Vetizou M., Prescott S.M., Fernandes M.R. (2022). Intestinal microbiota signatures of clinical response and immune-related adverse events in melanoma patients treated with anti-PD-1. Nat. Med..

[15] Cristescu R., Mogg R., Ayers M., Albright A., Murphy E., Yearley J., Sher X., Liu X.Q., Lu H., Nebozhyn M. (2019). Pan-tumor genomic biomarkers for PD-1 checkpoint blockade-based immunotherapy. Science.

[16] Komiya K., Nakamura T., Abe T., Ogusu S., Nakashima C., Takahashi K., Kimura S., Sueoka-Aragane N. (2019). Discontinuation due to immune-related adverse events is a possible predictive factor for immune checkpoint inhibitors in patients with non-small cell lung cancer. Thorac. Cancer.

[17] Bilger G., Girard N., Doubre H., Levra M.G., Giroux-Leprieur E., Giraud F., Decroisette C., Carton M., Massiani M.A. (2021). Discontinuation of immune checkpoint inhibitor (ICI) above 18 months of treatment in real-life patients with advanced non-small cell lung cancer (NSCLC): INTEPI, a multicentric retrospective study. Cancer Immunol. Immunother..

[18] Iivanainen S., Koivunen J.P. (2020). Possibilities of Improving the Clinical Value of Immune Checkpoint Inhibitor Therapies in Cancer Care by Optimizing Patient Selection. Int. J. Mol. Sci..

[19] Larkin J., Chiarion-Sileni V., Gonzalez R., Grob J.-J., Cowey C.L., Lao C.D., Schadendorf D., Dummer R., Smylie M., Rutkowski P. (2015). Combined Nivolumab and Ipilimumab or Monotherapy in Untreated Melanoma. N. Engl. J. Med..

[20] Powles T. (2018). Re: Nivolumab plus Ipilimumab Versus Sunitinib in Advanced Renal-cell Carcinoma. Eur. Urol..

[21] Wang Y., Wiesnoski D.H., Helmink B.A., Gopalakrishnan V., Choi K., DuPont H.L., Jiang Z.-D., Abu-Sbeih H., Sanchez C.A., Chang C.-C. (2018). Fecal microbiota transplantation for refractory immune checkpoint inhibitor-associated colitis. Nat. Med..

[22] Robinson I., Schmidinger M., Hochmair M., Ay L., Absenger G., Pichler M., Nguyen V., Richtig E., Rainer B., Jansen C. (2022). 117P BiomeOne: Multi-centric validation of a novel microbiome-based biomarker to predict response to cancer immunotherapy. Ann. Oncol..

[23] Robinson I., Hochmair M., Ay L., Absenger G., Jansen C., Pacifico C., Sladek B., Knabl A., Gasche N., Valipour A. (2022). 55P Comparison of BiomeOne and PD-L1 expression tests as a predictor for response to immune checkpoint inhibitors (ICI) in patients with non-small cell lung cancer (NSCLC). Ann. Oncol..

[24] Lopez-Siles M., Duncan S.H., Garcia-Gil L.J., Martinez-Medina M. (2017). *Faecalibacterium prausnitzii*: From microbiology to diagnostics and prognostics. ISME J..

[25] Cebula A., Seweryn M., Rempala G.A., Pabla S.S., McIndoe R.A., Denning T.L., Bry L., Kraj P., Kisielow P., Ignatowicz L. (2013). Thymus-derived regulatory T cells contribute to tolerance to commensal microbiota. Nature.

[26] Botticelli A., Putignani L., Zizzari I., Del Chierico F., Reddel S., Di Pietro F., Quagliarello A., Onesti C.E., Raffaele G., Mazzuca F. (2018). Changes of microbiome profile during nivolumab treatment in NSCLC patients. J. Clin. Oncol..

[27] Jin Y., Dong H., Xia L., Yang Y., Zhu Y., Shen Y., Zheng H., Yao C., Wang Y., Lu S. (2019). The Diversity of Gut Microbiome is Associated with Favorable Responses to Anti–Programmed Death 1 Immunotherapy in Chinese Patients with NSCLC. J. Thorac. Oncol..

[28] Zitvogel L., Ayyoub M., Routy B., Kroemer G. (2016). Microbiome and Anticancer Immunosurveillance. Cell.

[29] Liberini V., Mariniello A., Righi L., Capozza M., Delcuratolo M.D., Terreno E., Farsad M., Volante M., Novello S., Deandreis D. (2021). NSCLC Biomarkers to Predict Response to Immunotherapy with Checkpoint Inhibitors (ICI): From the Cells to In Vivo Images. Cancers.

[30] Haratani K., Hayashi H., Chiba Y., Kudo K., Yonesaka K., Kato R., Kaneda H., Hasegawa Y., Tanaka K., Takeda M. (2018). Association of Immune-Related Adverse Events with Nivolumab Efficacy in Non–Small-Cell Lung Cancer. JAMA Oncol..

[31] Lee K.A., Thomas A.M., Bolte L.A., Björk J.R., de Ruijter L.K., Armanini F., Asnicar F., Blanco-Miguez A., Board R., Calbet-Llopart N. (2022). Cross-cohort gut microbiome associations with immune checkpoint inhibitor response in advanced melanoma. Nat. Med..

