# Supplementary material for: Assessing the Performance of a Novel Stool-Based Microbiome Test That Predicts Response to First Line Immune Checkpoint Inhibitors in Multiple Cancer Types
Source: Cancers (Basel). 2023 Jun 21;15(13):3268. doi: 10.3390/cancers15133268 (PMC10339964; doi:10.3390/cancers15133268)
Supplement: Supplementary file 1 [file cancers-15-03268-s001.zip › Supplementary Data_corrected.pdf]

Figure S1. Flowchart of patients enrolled since study entry and details about inclusion and exclusion criteria. All analysis performed with this dataset are highlighted in orange and are accompanied by the respective number (*n*), which represents the total amount of patients included for each analysis step.

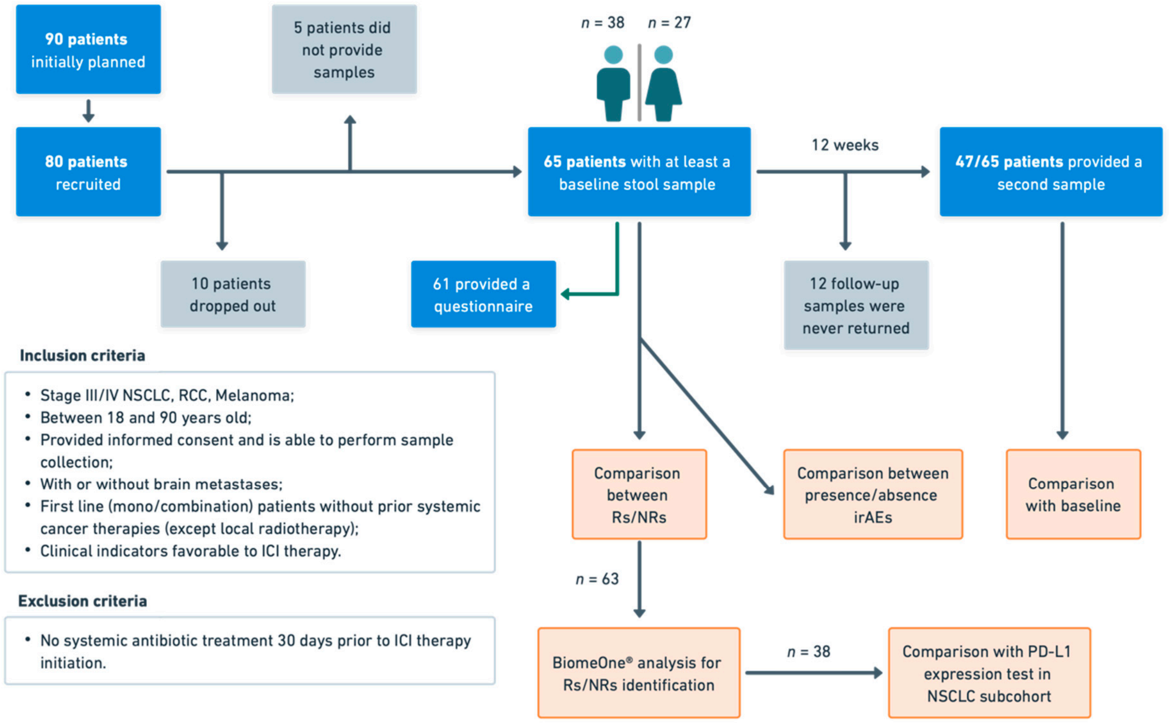

Table S1. Questionnaire information obtained during baseline sample collection. Results are given in numbers (*n*) and respective %, except when stated otherwise. Fisher's exact test was used to detect differences between Rs and NRs at a significance level of 0.05.

	**Total (*n* = 61)***	**R (*n* = 39)**	**NR (*n* = 22)**	***P*-value**
**Patients**				
**Weight (mean ± SD, kg)**	76.22 ± 15.66	77.57 ± 16.61	73.86 ± 13.90	0.34
**Height (mean ± SD, cm)**	171.5 ± 10.51	172.62 ± 10.37	169.52 ± 10.72	0.41
**BMI (mean ± SD, kg/m²)**	25.87 ± 4.66	25.98 ± 4.87	25.69 ± 4.39	0.70
**Ethnicity**				0.71
Asia	2 (3.28)	2 (5.13)	0 (0.00)	
Eastern Europe	8 (13.11)	4 (10.26)	4 (18.18)	
Western Europe	44 (72.13)	28 (71.79)	16 (72.73)	
Not disclosed	7 (11.48)	5 (12.82)	2 (9.09)	
**C-section**				0.21
Yes	3 (4.92)	2 (5.13)	1 (4.55)	
No	50 (81.97)	30 (76.92)	20 (90.91)	
Does not know	6 (9.84)	6 (15.38)	0 (0.00)	
Not disclosed	2 (3.28)	1 (2.56)	1 (4.55)	
**Allergies**				1.00
Yes	7 (11.48)	5 (12.82)	2 (9.09)	
No	54 (88.52)	34 (87.18)	20 (90.91)	
**Special diet**				0.79
Yes	3 (4.92)	2 (5.13)	1 (4.55)	
No	55 (90.16)	34 (87.18)	21 (95.45)	
Not disclosed	3 (4.92)	3 (7.69)	0 (0.00)	
**Gastrointestinal perturbations**[1]				0.23
No	32 (52.46)	22 (56.41)	10 (45.45)	
Diarrhea	1 (1.64)	1 (2.56)	0 (0.00)	
Constipation	2 (3.28)	0 (0.00)	2 (9.09)	
Flatulence/bloating	7 (11.48)	5 (12.82)	2 (9.09)	
Feeling full	0 (0.00)	0 (0.00)	0 (0.00)	
Stomach ache	2 (3.28)	1 (2.56)	1 (4.55)	
Heartburn	3 (4.92)	1 (2.56)	2 (9.09)	
Not disclosed	4 (6.56)	4 (10.26)	0 (0.00)	
More[2]	10 (16.39)	5 (12.82)	5 (22.73)	

**Alcohol consumption**				0.56
More often	4 (6.56)	3 (7.69)	1 (4.55)	
1-3 times per week	6 (9.84)	5 (12.82)	1 (4.55)	
Once a week	11 (18.03)	6 (15.38)	5 (22.73)	
Once a month	37 (60.66)	22 (56.41)	15 (68.18)	
Not disclosed	3 (4.92)	3 (7.69)	0 (0.00)	
**Smoking habits**				0.31
No	47 (77.05)	31 (79.49)	16 (72.73)	
Often	10 (16.39)	7 (17.95)	3 (13.64)	
Occasionally	4 (6.56)	1 (2.56)	3 (13.64)	
**Stress level**				0.60
Low (1-4)	30 (49.18)	19 (48.72)	11 (50.00)	
Medium (5-7)	15 (24.59)	8 (20.51)	7 (31.82)	
High (8-10)	14 (22.95)	10 (25.64)	4 (18.18)	
Not disclosed	2 (3.28)	2 (5.13)	0 (0.00)	
**Physical health**				0.34
Poor (1-4)	18 (29.51)	13 (33.33)	5 (22.73)	
Medium (5-7)	19 (31.15)	13 (33.33)	6 (27.27)	
Good (8-10)	20 (32.79)	10 (25.64)	10 (45.45)	
Not disclosed	4 (6.56)	3 (7.69)	1 (4.55)	
**Mental health**				0.21
Poor (1-4)	14 (26.23)	9 (23.08)	5 (22.73)	
Medium (5-7)	27 (44.26)	20 (51.28)	7 (31.82)	
Good (8-10)	17 (27.87)	8 (20.51)	9 (40.91)	
Not disclosed	3 (4.92)	2 (5.13)	1 (4.55)	

[*]4 patients did not complete the self-reported survey. [1]Patients that reported experiencing just one of these perturbations. [2]Other patients reported experiencing several gastrointestinal perturbations simultaneously, such as flatulence/bloating and heartburn (*n* = 4), diarrhea and heartburn (*n* = 1), diarrhea, constipation and stomachache (*n* = 1), constipation and flatulence bloating (*n* = 1), diarrhea, constipation, flatulence/bloating, feeling full and heartburn (*n* = 1), constipation, stomachache and heartburn (*n* = 1) and constipation, flatulence/bloating, feeling full and stomachache (*n* = 1).

Table S2. Questionnaire results regarding baseline sample information. Fisher's exact test was used to detect differences between Rs and NRs at a significance level of 0.05.

	**Total (*n* = 61)***	**R (*n* = 39)**	**NR (*n* = 22)**	***P*-value**
Stool				
**Consistency**				0.73
1	7 (11.48)	3 (7.69)	4 (18.18)	
2	6 (9.84)	2 (5.13)	1 (4.55)	
3	6 (9.84)	4 (10.26)	2 (9.09)	
4	20 (32.79)	13 (33.33)	7 (31.82)	
5	8 (13.11)	4 (10.26)	4 (18.18)	
6	2 (3.28)	1 (2.56)	1 (4.55)	

Not disclosed	12 (19.67)	9 (23.08)	3 (13.64)	
**Frequency**				0.27
More often	1 (1.64)	1 (2.56)	0 (0.00)	
1-3 times per day	36 (59.02)	26 (66.67)	10 (45.45)	
Every 2 days	19 (31.15)	10 (25.64)	9 (40.91)	
Less often	4 (6.56)	2 (5.13)	2 (9.09)	
Not disclosed	1 (1.64)	0 (0.00)	1 (4.55)	

*4 patients did not complete the self-reported survey.

Table S3. Differentially abundant (DA) microbial species identified by ALDEx2 between Rs and NRs classified by the BiomeOne® algorithm. ALDEx2 uses the Wilcoxon Rank Sum test and Welch's t-test to analyze clr-transformed variation in taxa between samples. A total of 7 DA species could be identified using this method.

**ALDEx2**	**Welch's t test (*P*-value)**	**Wilcoxon rank test (*P*-value)**	**Median clr value NRs**	**Median clr value Rs**	**Median effect size**
Oscillospira sp.	< 0.05	< 0.05	0.97	6.19	0.58
Sutterella sp.	0.01	0.01	9.16	2.85	-0.51
Streptococcus sp.	0.02	0.05	8.03	6.72	-0.31
(order) Clostridia UCG-014	0.02	0.01	2.16	8.13	0.43
Prevotella copri	0.04	0.08	1.88	4.25	0.28
Lachnospiraceae UCG-010 sp.	0.06	0.04	4.04	5.58	0.33
(order) Lactobacillales	0.07	0.03	3.56	1.58	-0.35

Table S4. Differentially abundant (DA) microbial species identified by MaAsLin2 between Rs and NRs classified by the BiomeOne® algorithm. MaAsLin2 performs general linear models on clr-transformed species abundance data, identifying a total of 21 DA taxa in our dataset.

**MaAsLin2**	**Coefficient***	**Standard error**	***P*-value**
Oscillospira sp.	1.93	0.52	< 0.01
Sutterella sp.	-2.54	0.83	< 0.01
Paludicola sp.	-0.95	0.33	0.01
Shuttleworthia sp.	-0.83	0.31	0.01
(order) Clostridia UCG-014	1.99	0.80	0.02
(family) Clostridiaceae	-0.91	0.37	0.02
(order) Lactobacillales	-0.85	0.35	0.02

Parasutterella secunda	-0.87	0.36	0.02
Bacteroides finegoldii	-1.69	0.71	0.02
Romboutsia sedimentorum	-0.74	0.31	0.02
Gemella sanguinis	-0.50	0.22	0.02
Streptococcus sp.	-1.21	0.54	0.03
Lachnospiraceae UCG-010 sp.	1.13	0.51	0.03
Prevotella copri	1.82	0.83	0.03
CHKCI002 sp.	-0.37	0.17	0.03
Lachnospiraceae NC2004 group sp.	1.56	0.73	0.04
Coprobacillus cateniformis	0.81	0.38	0.04
Bacteroides coprocola	-1.33	0.63	0.04
Methanobrevibacter sp.	-0.86	0.41	0.04
Bifidobacterium bifidum	-0.85	0.42	0.05
(family) UCG-010	1.25	0.62	0.05

*Reference level used was the Rs group. Negative coefficients indicate the microbial species that decreased in Rs, while positive coefficients indicate microbial species enriched in Rs.
